# Association between living arrangements and cognitive decline in older adults: A nationally representative longitudinal study in China

**DOI:** 10.1186/s12877-022-03473-x

**Published:** 2022-11-08

**Authors:** Yifan Yu, Junqi Lv, Jing Liu, Yueqiao Chen, Kejin Chen, Yanfang Yang

**Affiliations:** Department of Epidemiology and Biostatistics, West China School of Public Health and West China Fourth Hospital, Sichuan University, No.17 Section 3, Renmin South Road, 610041 Chengdu, Sichuan China

**Keywords:** Living arrangements, Older adults, Cognitive decline, Multilevel modelling, Longitudinal study

## Abstract

**Background:**

Living arrangements are critical to the survival and well-being of older people, especially in China where the filial piety culture demands adult children care for and serve their parents. The study aimed to explore the association between living arrangements and cognitive decline among older people in China.

**Methods:**

Participants included 6,074 older adults over 60 years old (49.65% male, mean age 67.2 years [range 60–98]) from four waves (2011–2018) of the China Health and Retirement Longitudinal Study. Two to four assessments were conducted over a follow-up of an average of 5.3 years (range, 2–7). Cognitive function was assessed using an adapted Chinese version of Mini-Mental State Examination (MMSE). Living arrangements were classified as follows: living alone, living with spouse, living with adult children, living with spouse and adult children and living with others. Multilevel models were used to investigate the relationship between living arrangements and cognitive decline, as well as the gender difference.

**Results:**

As the main type of living arrangements of the study participants (44.91%), living with spouse was taken as the reference group. Compared to the reference group, living alone (*β*=-0.126, *P* < 0.001), living with adult children (*β*=-0.136, *P* < 0.001), living with spouse and adult children (*β*=-0.040, *P* < 0.05) and living with others (*β*=-0.155, *P* < 0.05) were all related to a faster rate of cognitive decline. Further, the association between living arrangements and cognitive decline varied by gender. Living alone (*β*=-0.192, *P* < 0.001) was associated with a faster cognitive decline only in older men. Living with spouse and adult children (*β*=-0.053, *P* < 0.05) and living with others (*β*=-0.179, *P* < 0.05) were associated with faster cognitive decline only in older women.

**Conclusion:**

This study suggests that living arrangements in older people in China were associated with cognitive decline, and these associations varied by gender. Greater attention to living arrangements might yield practical implications for preserving the cognitive function of the older population.

**Supplementary Information:**

The online version contains supplementary material available at 10.1186/s12877-022-03473-x.

## Background

A large part of the global population is aging, and the rate at which this occurs is accelerating, especially in China [1]. According to the 2020 Population Census of China, the number of people aged 60 and older has reached 263 million, and its proportion of the overall population has risen from 13.26% to 18.70% in the last decade [2]. Dementia is one of the most common and serious disorders in the aged population. The ageing of the world’s population makes dementia a global public health problem. According to the World Alzheimer report 2021, over 55 million people were suffering from dementia in the world, and this figure is set to reach 78 million by 2030 [3]. During the long preclinical phase of dementia, cognitive decline is considered to be one of the early risk factors and defining features of Alzheimer’s disease (AD) and other dementias [4–7].In addition, older people with cognitive decline are more likely to engage in limited daily living activities and require constant care from their families and society [8], which could have a negative effect on functioning, quality of life, the load on family caregivers, and the cost of medical care [9]. Therefore, in order to delay or prevent dementia and reduce the socioeconomic burden, the recognition of possibly modifiable risk factors of rapid cognitive decline is of vital importance.

Living arrangement is critical for the psychological well-being of older adults because they provide an invaluable network of social support. Conform to the Confucian principle of filial piety, traditional Chinese values greatly emphasize the interdependence between parents and children and encourage them to live under one roof [10]. However, the economic revolution and growing urbanization as well as the strict family planning policy in the late 1970s have dramatically transformed traditional family structures and living arrangements in China [11]. An increasing number of older Chinese people are actively or passively choosing to live with their spouse or alone rather than with their adult children [12, 13]. This change has raised concerns about the reliability of the support for older adults from families in China.

The current evidence on the associations between living arrangements and cognition function remains mixed. A large body of the literature suggests that living with household members is more advantageous than living alone for mental health as well as maintaining cognitive function [14, 15]. Older people who live alone were associated with higher loneliness, slower processing speed and higher risk of depression [16, 17]. Conversely, other studies have found that older adults living with household members had higher risk of cognitive impairment, compared to those living alone [12, 18]. Specifically, older adults who live with their children were disadvantaged in social, economic and mental well-being and were more likely to be disabled [19, 20]. They also had a greater risk of cognitive impairment and higher dementia severity [12, 21]. However, most of the aforementioned studies were cross-sectional or conducted in developed countries (England, Canada and Singapore etc.). It is still necessary to further explore the longitudinal relationship between living arrangements and cognitive decline in a large community-dwelling elderly sample in China.

Unlike developed countries, developing and emerging countries in Asia (especially China) widely retain traditional family norms, such as filial piety, and have inadequate public pension and social security systems [14, 22]. The family is still the primary source of support for older adults in China. In other words, living with household members may be beneficial for the health of older Chinese people. Therefore, we hypothesize that older Chinese adults living with their spouses and (or) children have slower cognitive decline.

We also hypothesize that males and females differ in the association of living arrangements and cognitive decline, because of their different social expectations, functions and family roles over a lifetime [23]. In traditional marriage, Chinese women tend to be more involved in household activities, while men are more dependent on their spouses to handle their lives, such as housework and daily care [24]. Thus, we expect living alone would have fewer adverse impacts on cognitive decline in women than in men. Furthermore, based on the traditional Chinese parent-child relationship, men tend to appear as “strict fathers”, which leads to certain barriers for men to readily express their feelings and build intimate relationships with their children [25]. We also expect that living with children is more beneficial to older women’s cognitive function than to men’s.

In this study, we wanted to determine the effects of living arrangements on cognitive decline among older Chinese adults. More explicitly, this study aimed to address the following: whether living alone versus with family member(s) is associated with cognitive decline and if there is a gender difference in this association. We hope our research could provide some recommendations for protecting the cognitive function of the old population and offer some policy references for developing a more appropriate aged care system in China.

## Methods

### Study design and participants

This study used data from four waves of the China Health and Retirement Longitudinal Study (CHARLS 2011–2018), a nationally representative longitudinal survey of the residents in China 45 years of age and above along with their spouses. To achieve sample representativeness, a multistage probability sampling approach was used. In 2011, respondents were interviewed face to face, and they were followed up in 2013, 2015, and 2018. The response rate for the baseline survey was 80.5%, and the follow-up response rates for 2011, 2013 and 2018 were 82.6%, 82.1% and 83.8% respectively [26]. CHARLS was authorized by Peking University’s Ethical Review Committee (IRB00001052-11014). Prior to participation, each participant signed an informed consent form. The cohort profile literature contains detailed explanations of the survey design and processes used in the CHARLS [27].

**Fig. 1:**
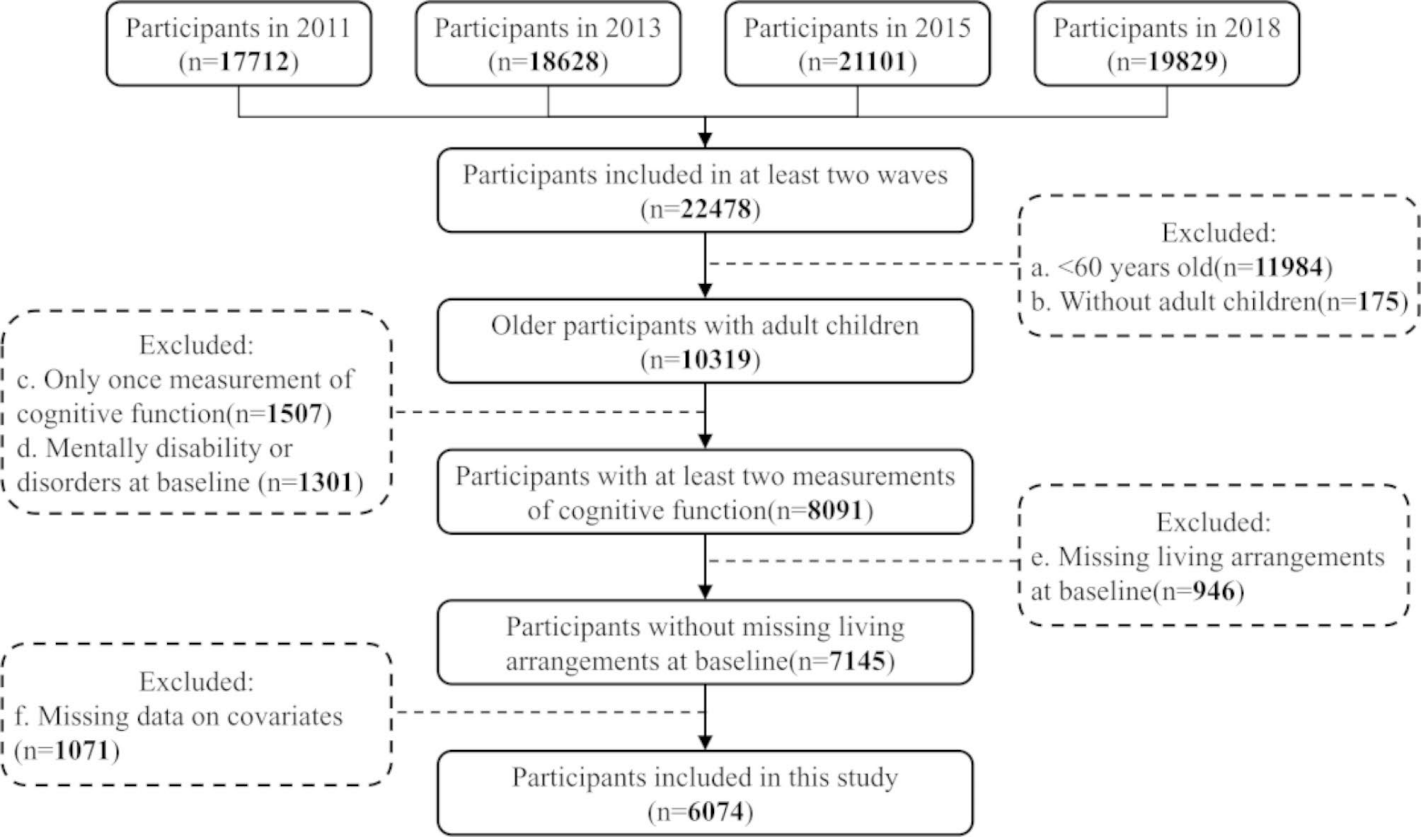
Recruitment flow chart

In this study, our analysis was limited to participants aged 60 and over. To take full advantage of data from four follow-ups, individuals who had reached the age of 60 and above during the study period (2011–2018) and had at least two (range 2–4) cognitive function assessments after 60 were eligible for inclusion. The participant’s first interview record was considered as the baseline (i.e., wave 1, 2, or 3, depending on when they joined CHARLS). For analytical purposes, we excluded: (1) participants without adult children (adult children were defined as children age 22 or older who were not schooled at that time); (2) participants with missing information on living arrangements at baseline; (3) participants with brain-damaged, mentally deficient, psychiatric problems or memory-related disorders at baseline; and (4) participants with missing information on demographic characteristics (gender, geographic residence, education, working status), health status (physical comorbidity, feeling pain, instrumental activities of daily living [IADLs], depressive symptoms, social activity participation), child characteristics (the number of adult children, average years of schooling of adult children), and socioeconomic level (average annual household expenditure per capita). The final sample is comprised of 6074 older respondents without missing key variables. We compared the baseline characteristics between participants included in the final analysis and the others who were excluded due to data missing (Table S2 in the Supplementary Material). The recruitment flow chart of the current study is indicated in Fig. 1.

### Measures

#### Cognitive function

Consistent with previous studies [28–31], cognitive function was captured using three categories: mental status, visuo-construction and episodic memory. Questions about orientation and numeric ability were used in CHARLS to measure mental status. Orientation was measured by asking respondents to identify the date (month, day, year), season, and day of the week. Numeric ability was measured by serial subtraction of 7 from 100 (up to five times). Based on the number of correct answers, scores on these questions were summed into the mental status score and ranged from 0 to 10. The score of the visuo-construction was recorded as 1 if the participants could replicate a figure previously displayed; otherwise, it was recorded as 0. Episodic memory was evaluated by a word recall test. Participants were asked to recall as many of the 10 unrelated Chinese words they had just heard as they could (immediate recall). Five minutes later, they were tasked with recalling the identical list of words (delayed recall) [30]. Episodic memory scores were calculated as the average score for immediate and delayed word recalls, ranging from 0 to 10.

The cognitive function scores varied from 0 to 21 and were calculated as the total of the mental status, visuo-construction and episodic memory scores. The higher the score, the better the cognitive function. The Cronbach’s alpha is 0.78 [32], which shows a satisfactory level of internal consistency.

#### Living arrangements

To fully explicate important details concerning different types of living arrangements and answer the research hypothesis, living arrangements were divided into the following five mutually exclusive categories based on the baseline survey: (A) living alone. (B) living with spouse (no adult children, may have others). (C) living with adult children (no spouse, may have others). (D) living with both spouse and adult children (may have others). (E) living with others who are not spouse or children.

#### Covariates

Given that cognitive function and living arrangements may differ depending on demographic characteristics, health status, child characteristics, and socioeconomic level, the following variables were included in this study as covariates. A time variable was also included that accounted for the number of years elapsed since the baseline interview.

Demographic characteristics included age (at baseline), gender (male or female), geographic residence (urban or rural), education (no formal education, capable of reading and/or writing, primary school, middle school and above) and working status (yes or no).

Health status was measured according to physical comorbidity, feeling pain (yes or no), instrumental activities of daily living (IADLs) (impaired or unimpaired), depressive symptoms (yes or no), and social activity participation (yes or no). Physical comorbidity data included conditions for which respondents self-reported receiving a diagnosis from a physician, such as dyslipidemia, diabetes or high blood sugar, chronic lung disease, etc. The number of physical comorbidities was calculated and categorized as 0,1–2 and ≥ 3. Feeling pain was self-reported via a question: “Are you often troubled with any body pains?”. IADLs were evaluated by the Lawton and Brody’s scale referring to doing housework, cooking, taking medicine, shopping, and taking care of finances [33]. Participants who reported having any difficulty in any items were classified as with IADLs impaired [34]. The Chinese version of the 10-item Center for Epidemiologic Studies Depression (CESD-10) Scale was used to measure depressive symptoms, which reflected the respondents’ depressive symptoms experienced over the last week. The ten items included three items on depressed mood, five items on somatic symptoms and two items on positive mood. Except for two items on positive emotions which were reverse-scored, the other eight items were scored 0,1,2,3 according to their frequency of symptoms. The total CESD-10 score for the 10 items ranges from 0 to 30, with higher scores indicating more severe depressive symptoms. Participants with a CESD-10 score above 10 points were sorted as depressed [35]. The CHARLS questionnaire included eleven categories of social activities. Participation in social activities was defined as the respondent having participated in at least one of these social activities in the last month.

Child characteristics included the number of adult children of respondents and average years of schooling of adult children which was defined to assess the overall educational attainment of adult children. The number of adult children was classified into three categories: 1, 2–3 and ≥ 4. The average years of schooling of adult children was centered by subtracting the mean value.

Following previous studies [36, 37], we calculated the average annual household expenditure per capita to measure the household resources. In developing countries, expenditure is a better way to assess the economic resources available to households than income. The measurement of expenditure also has less error than income. To capture the non-linear relationship between income and outcome variables, the average annual household expenditure was log-transformed in the analysis.

Additional details of covariates are available in supplemental Methods and Table S1 in the Supplementary Material.

### Statistical analysis

We used descriptive statistics to describe the characteristics of respondents. Continuous variables were presented as the mean and standard deviation (SD). Categorical variables were presented as frequency (n) and percentage (%). t-test and chi-square test were used to identify significant differences in characteristics between males and females.

Multilevel models were used to assess the relationships between cognitive decline and living arrangements. Multilevel models are the optimal approach for analyzing nested data that are not independently observed (e.g. time points within individuals) and contradict the assumption of independent observations [38]. An important advantage of multilevel growth models is that they can handle unbalanced data, which means that they do not require the same number of measurement occasions per individual to obtain efficient estimates [39]. In this study. The data structure was that up to four waves of repeat measurement data (level 1) were nested within 6074 individuals (level 2).

Using data from up to four waves of data collection, we estimated three models for all respondents first and then separately for males and females. Model 1: adjusted model containing time, living arrangement and part of the covariates (age, gender and geographic residence [urban or rural]). Model 2: adjusted model containing time, living arrangement and all of the covariates (age, geographic residence, education, working status, physical comorbidities, feeling pain, IADLs, depressive symptoms, social activity participation, number of adult children, average schooling year of children, household expenditure per capita). Model 3: add the interacting term of living arrangement and time to Model 2. It was built to address our hypotheses concerning the association between living arrangements and cognitive decline. Older people living with their spouse was regarded as the reference group. The differences in the rate of cognitive decline between living with spouse and other types of living arrangements were indicated by the regression coefficients of living arrangements × time. To examine potential gender-specific effects, a stratified analysis by gender was conducted. Living arrangements and all covariates from baseline evaluation were treated as time-invariant.

Considering that a large proportion of participants were excluded due to missing data, a sensitivity analysis was performed using multiple imputation (multilevel joint modelling multiple imputation) [40]. The results were similar after multiple imputation (shown in Table S3 and S4 in the Supplementary Material). We did not apply sampling weights in present study. Because the use of sampling weights in estimating causal effects and multilevel analysis is controversial and ambiguous [41–45]. And several studies using CHARLS data have shown that the results of weighted and unweighted analyses were similar [46, 47] .

All descriptive analyses were conducted using STATA version 16.0 software, and multilevel analyses were performed using MLwiN 2.30 software. *P* < 0.05 was regarded as statistically significant.

## Results

The results of descriptive statistics are presented in Table 1. A total of 6074 participants were included in our final analyses (49.65% male), with an average age of 67.24 ± 0.08 years old at baseline. Of all respondents, the score of cognitive function at baseline was 9.93 ± 4.26, and it declined at each follow-up survey. The largest proportion of older people lived with their spouse (44.91%), followed by those who live with their spouse and adult children (34.62%). The proportion of older people who lived alone was 7.94%. As a result of the gender comparison, cognitive function scores were higher among male respondents. Male respondents were also more likely to live with their spouse or live with both spouse and adult children. In addition, male respondents were more likely to be highly educated, still work, be in better health, have fewer adult children and have more educated adult children than females.

**Table 1:** Characteristics of study participants

	All (n = 6074)	Male (n = 3016)	Female (n = 3058)	*P* value of gender difference
**Cognitive function, mean ± SD**				
2011	10.04 ± 4.13	11.18 ± 3.68	8.89 ± 4.24	< 0.001
2013	9.56 ± 4.51	10.82 ± 4.04	8.31 ± 4.60	< 0.001
2015	9.03 ± 4.58	10.26 ± 4.10	7.81 ± 4.70	< 0.001
2018	8.91 ± 4.89	9.97 ± 4.36	7.76 ± 5.11	< 0.001
**The living arrangement, n (%)**				< 0.001
Living alone	482 (7.94)	172 (5.70)	310 (10.14)	
Living with spouse	2728 (44.91)	1443 (47.84)	1285 (42.02)	
Living with adult children	639 (10.52)	186 (6.17)	453 (14.81)	
Living with spouse and adult children	2103 (34.62)	1169 (38.76)	934 (30.54)	
Living with others	122 (2.01)	46 (1.53)	76 (2.49)	
**Age (years at baseline), mean ± SD**	67.24 ± 5.99	67.30 ± 5.90	67.18 ± 6.07	0.410
**Geographic residence, n (%)**				0.208
Rural	3627 (59.71)	1825 (60.51)	1802 (58.93)	
Urban	2447 (40.29)	1191 (39.49)	1256 (41.07)	
**Education, n (%)**				< 0.001
No formal education	1729 (28.47)	382 (12.67)	1347 (44.05)	
Capable of reading and/or writing	1300 (21.40)	640 (21.22)	660 (21.58)	
Primary school	1600 (26.34)	1004 (33.29)	596 (19.49)	
Middle school and above	1445 (23.79)	990 (32.82)	455 (14.88)	
**Working status, n (%)**				< 0.001
No	2710 (44.62)	1138 (37.73)	1572 (51.41)	
Yes	3364 (55.38)	1878 (62.27)	1486 (48.59)	
**The number of physical comorbidities, n (%)**				< 0.001
0	1858 (30.59)	1000 (33.16)	858 (28.06)	
1–2	2972 (48.93)	1470 (48.74)	1502 (49.12)	
≥ 3	1244 (20.48)	546 (18.10)	698 (22.83)	
**Feeling pain, n (%)**				< 0.001
No	4050 (66.68)	2233 (74.04)	1817 (59.42)	
Yes	2024 (33.32)	783 (25.96)	1241 (40.58)	
**IADLs, n (%)**				< 0.001
Unimpaired	4630 (76.23)	2452 (81.30)	2178 (71.22)	
Impaired	1444 (23.77)	564 (18.70)	880 (28.78)	
**Depressive symptoms, n (%)**				< 0.001
No	3854 (63.45)	2134 (70.76)	1720 (56.25)	
Yes	2220 (36.55)	882 (29.24)	1338 (43.75)	
**Social activity participation, n (%)**				0.238
No	3198 (52.65)	1565 (51.89)	1633 (53.40)	
Yes	2876 (47.35)	1451 (48.11)	1425 (46.60)	
**Number of adult children, n (%)**				< 0.001
1	582 (9.58)	340 (11.27)	242 (7.91)	
2–3	3041 (50.07)	1588 (52.65)	1453 (47.51)	
≥ 4	2451 (40.35)	1088(36.07)	1363 (44.57)	
**Average schooling year of children (Centered), mean ± SD**	8.41 ± 3.60	8.58 ± 3.62	8.23 ± 3.57	< 0.001
**Household expenditure per capita(log), mean ± SD**	8.54 ± 0.94	8.57 ± 0.93	8.52 ± 0.96	0.018

Note: (1) Except for the variable cognitive function, all other variables were measured at baseline interview. (2) Sex differences in continuous variables were tested using t-tests and in categorical variables using chi-square tests. (3) The number of observations in 2011, 2013, 2015 and 2018 were 4757, 5400, 5434 and 3714 respectively. The total number of observations was 19,305 with 6074 individuals. (4) The results were unweighted. (5) SD = standard deviation, IADLs = instrumental activity of daily living

The results of the multilevel growth model fit for Models 1–3 are listed in Table 2. Model 1–2 showed the relationship between living arrangements and cognitive function status. In Model 1, both time and the living arrangements were associated with cognitive function significantly after controlling for age, gender and geographic residence. On average, scores for cognitive function have decreased by 0.085 units per year. In contrast to older people living with their spouse, the scores of cognitive function were lower among older people living alone, living with adult children, living with spouse and adult children, and living with others, by 0.499, 1.232, 0.544 and 1.345 respectively. After controlling for all covariates in Model 2, the time term was still negative (*β*=-0.086, *P* < 0.001). The association between a part of living arrangements and cognitive function remained significant but was slightly attenuated. In comparison to older adults living with their spouse, individuals living with their adult children had a higher risk of having poorer cognitive function (*β*=-0.405, *P* < 0.01). Estimate of the regression coefficient of gender (*β*=-0.689, *P* < 0.001) was negative indicating a worse cognitive function state for female older adults.

**Table 2:** The association between living arrangements and cognitive function

	Model 1	Model 2	Model 3
**Fixed Effects**			
Intercept	11.291^***^	5.582^***^	5.489^***^
Living arrangement (ref = Living with spouse)			
Living alone	-0.499^**^	-0.180	0.144
Living with adult children	-1.232^***^	-0.405^**^	-0.055
Living with spouse and adult children	-0.544^***^	-0.108	-0.002
Living with others	-1.345^***^	-0.246	0.159
Time (years since baseline)	-0.085^***^	-0.086^***^	-0.048^***^
Age (ref = 60–64, at baseline)			
65–69	-0.689^***^	-0.465^***^	-0.469^***^
70–74	-1.806^***^	-1.067^***^	-1.068^***^
≥ 75	-3.334^***^	-1.839^***^	-1.845^***^
Gender (ref = Male)			
Female	-2.348^***^	-0.698^***^	-0.695^***^
Geographic residence (ref = Rural)			
Urban	2.539^***^	0.445^***^	0.444^***^
Education (ref = No formal education)			
Capable of reading and/or writing		2.435^***^	2.434^***^
Primary school		4.030^***^	4.030^***^
Middle school and above		5.152^***^	5.154^***^
Working status (ref = No)			
Yes		0.009	0.015
The number of physical comorbidities (ref = 0)			
1–2		0.026	0.026
≥ 3		0.176	0.177
Feeling pain (ref = No)			
Yes		-0.181^*^	-0.182^*^
IADLs (ref = Unimpaired)			
Impaired		-0.801^***^	-0.803^***^
Depressive symptoms (ref = No)			
Yes		-0.485^***^	-0.487^***^
Social activity participation (ref = No)			
Yes		0.578^***^	0.577^***^
Number of adult children (ref = 1)			
2–3		0.185	0.183
≥ 4		0.103	0.101
Average years of schooling of adult children (Centered)		0.203^***^	0.203^***^
Household expenditure per capita (log)		0.259^***^	0.258^***^
Time * Living alone			-0.126^***^
Time * Living with adult children			-0.136^***^
Time * Living with spouse and adult children			-0.040^*^
Time * Living with others			-0.155^*^
**Random Effects**			
Level 2: Individual			
Individual -variance	9.991^***^	4.695^***^	4.703^***^
Level 1: Point in time			
Point in time-variance	6.522^***^	6.504^***^	6.486^***^
-2*loglikelihood	101598.05	98079.63	98042.90

Note: (1) Figures in the table were parameter estimates based on unweighted multilevel models from 6074 individuals consisting of 19,305 observations. (2) Model estimation was based on the iterative generalized least squares. (3) ref = the reference category, IADLs = instrumental activity of daily living. (4) ^*^P < 0.05, ^**^P < 0.01, ^***^P < 0.001

Model 3 examined the differences in the rates of cognitive decline between older people with different living arrangements by adding the interacting terms of living arrangement and time to Model 2. The coefficient on the interaction terms was statistically significant. In comparison to older people living with their spouse, those who live alone (*β*=-0.126, *P* < 0.001), live with adult children (*β*=-0.136, *P* < 0.001), live with spouse and adult children (*β*=-0.040, *P* < 0.05) and who live with others (*β*=-0.155, *P* < 0.05) were significantly associated with a faster rate of cognitive decline after controlling for covariates. The coefficient for *gender* (*β*=-0.695, *P* < 0.001) remained statistically significant after the inclusion of the interaction term, suggesting that females had worse cognitive function. Models 2–3 have lower - 2 log-likelihood values than Model 1, indicating a better fit.

Table 3 presents the results of the gender-stratified analysis. The relationship between living arrangements and cognitive decline was significant in both males and females, whereas there was a gender difference. In comparison to living with spouse, living with adult children was related to faster cognitive decline for both male (*β*=-0.168, *P* < 0.01) and female (*β*=-0.107, *P* < 0.01) older people. Living alone (*β*=-0.192, *P* < 0.001) was related to faster cognitive decline only in older men. Living with spouse and adult children (*β*=-0.053, *P* < 0.05), as well as living with others (*β*=-0.179, *P* < 0.05), was associated with a faster rate of cognitive decline in older women, but not with the rate of cognitive decline in older males.

**Table 3:** Stratification analysis of the association between living arrangements and cognitive decline

	Males (n = 3016)	Females (n = 3058)
**Fixed Effects**		
Intercept	4.634^***^	6.000^***^
Living arrangement (ref = Living with spouse)		
Living alone	0.004	0.159
Living with adult children	-0.037	-0.126
Living with spouse and adult children	0.065	-0.084
Living with others	-0.019	0.182
Time (years since baseline)	-0.021	-0.077^***^
Age (ref = 60–64, at baseline)		
65–69	-0.293^**^	-0.673^***^
70–74	-1.034^***^	-1.165^***^
≥ 75	-1.818^***^	-1.940^***^
Geographic residence (ref = Rural)		
Urban	0.236^*^	0.591^***^
Education (ref = No formal education)		
Capable of reading and/or writing	2.341^***^	2.274^***^
Primary school	3.660^***^	4.179^***^
Middle school and above	4.800^***^	5.398^***^
Working status (ref = No)		
Yes	0.060	-0.081
The number of physical comorbidities (ref = 0)		
1–2	0.146	-0.066
≥ 3	0.182	0.156
Feeling pain (ref = No)		
Yes	-0.283^*^	-0.103
IADLs (ref = Unimpaired)		
Impaired	-0.837^***^	-0.782^***^
Depressive symptoms (ref = No)		
Yes	-0.460^***^	-0.477^***^
Social activity participation (ref = No)		
Yes	0.656^***^	0.510^***^
Number of adult children (ref = 1)		
2–3	0.249	0.022
≥ 4	0.231	-0.089
Average schooling year of children (Centered)	0.174^***^	0.237^***^
Household expenditure per capita (log)	0.360^***^	0.154^**^
Time * Living alone	-0.192^***^	-0.071
Time * Living with adult children	-0.168^**^	-0.107^**^
Time * Living with spouse and adult children	-0.032	-0.053^*^
Time * Living with others	-0.061	-0.179^*^
**Random Effects**		
Level 2: Individual		
Individual -variance	4.011^***^	5.274^***^
Level 1: Point in time		
Point in time-variance	6.795^***^	6.135^***^
-2*loglikelihood	49282.55	48645.26

Note: (1) Figures in the table were parameter estimates based on unweighted multilevel models from 3106 males consisting of 9699 observations and 3058 females consisting of 9606 observations. (2) Model estimation was based on the iterative generalized least squares. (3) ref = the reference category, IADLs = instrumental activity of daily living. (4) ^*^P < 0.05, ^**^P < 0.01, ^***^P < 0.001

## Discussion

Using four waves of China Health and Retirement Longitudinal Study(CHARLS) data, we investigated the associations between living arrangements and cognitive decline in older people in China. The gender-related difference in these associations was further examined. We found that participants living alone, living with adult children, living with spouse and adult children and living with others all had a faster rate of cognitive decline than those living with spouse. In addition, stratification to gender showed that living alone was related to faster cognitive decline only for male older people. Only in females was living with spouse and adult children or with others related to a faster rate of cognitive decline.

Findings from this study support part of our first hypothesis that older Chinese adults living with their spouse have slower cognitive decline. In China, the proportion of older people who lived with spouse had increased sharply, while the proportion of older people living with adult children had decreased substantially, due to increased preference for independent living, mobility of their offspring, lower mortality rates of their spouses, and higher remarriage rates among older persons [48]. Our study found that all four other types of living arrangements were associated with faster cognitive decline in comparison to living with spouse, suggesting that living with spouse may be a better choice for maintaining cognitive function. Additionally, the finding supports the assertion that having a spouse provides the “best guarantee of support in old age” [49]. Spouse may provide emotional support and intimate interaction to reduce the psychological stress and loneliness of older people, thereby slowing cognitive decline. Spouse also extends the older person’s personal network by connecting with people such as the spouse’s friends and family. Social engagement and a larger social network size could increase cognitive reserve and prevent cognitive decline [50, 51].

In comparison to older adults living with a spouse, those living alone experienced a faster rate of cognitive decline. Previous studies suggested those who live alone were more likely to experience greater social isolation and smaller social networks, which are predictive of a negative impact on cognitive function [16, 52]. Living alone also means that this group of older adults had experienced the loss of a spouse. Losing a spouse through widowhood, divorce, or separation is an important stressful life event in old age, and it has a detrimental influence on cognitive performance in older persons [53, 54].

It is worth noting that in our study, we observed that older people living with adult children or with both spouse and adult children experienced a faster rate of cognitive decline than those who lived alone, which was not consistent with our first hypothesis. Traditional Confucian values emphasize family and filial piety, and the practice of adult children living with their parents can be considered a part of filial piety. However, our finding indicates that living with adult children, as a traditional Chinese family concept, may not be conducive to the cognitive health of older people. The possible negative effects of living with children include the following. Although adult children may receive satisfaction in performing the duty of caring for their parents, they may also become physically and/or mentally stressed [55, 56], which can result in a high level of intergenerational conflict. In turn, this leads to cognitive decline in older people who received care from their children. Moreover, living with adult children may lead to an over-reliance on them for emotional or financial support, which can increase feelings of worthlessness and lead to impaired cognitive abilities [10]. Influenced by traditional Chinese culture, people value interdependence within the family and most older people will stay with their adult children and provide the necessary support when asked to do so by their adult children [57]. The burdens of helping their adult children during cohabitation might create long-term chronic stress. A study found that increasing levels of perceived stress were associated with worse initial cognitive status and a faster rate of cognitive decline among adults age 65 and over [58].

Our second hypothesis that males and females differ in the association of living arrangements and cognitive decline has also received some support from the results. The stratified analysis revealed that the association between cognitive decline and living arrangements varied by gender. In the present study, for men, living alone and living with adult children were associated with cognitive decline. For women, cognitive decline was associated with living with adult children, living with spouse and adult children and living with others, but not related to living alone. Women are usually in charge of household affairs and family activities and are more likely to provide physical care and emotional support to their spouses, which could protect the cognitive function of their male partners [59–61]. Correspondingly, women may be able to live alone in old age as a result of their life experience managing a household. Also, women tend to enjoy more extensive social networks than men through their participation in social activities and intimate friendships [62, 63], which likely compensates for the loneliness and the lack of intimacy of older women living alone.

The contribution of this study to the literature includes the following four areas. First, our study provides additional evidence for research related to cognitive function in older people in developing countries by focusing on the association between living arrangements and cognitive decline in older people in China. Second, using longitudinal data and multilevel modelling, our study is prospective, reducing the possibility of reverse causation bias. Third, our study used a national representative sample and therefore the findings are more generalizable. Last but not least, our study paid attention to the gender difference in cognitive decline.

Limitations of this study should also be noted. First, we used baseline living arrangements and covariates to determine the relationship between living arrangements and cognitive decline. Considering that the living arrangements of older people may change over time, further research needs to take into account the influence of time-varying living arrangements on cognitive function. Second, detailed information on the living arrangements of older people, such as the duration of current living arrangements, is not available, which also played an important role in exploring the relationship between living arrangements and cognitive decline in older people. Third, participants with missing key variates and less than four times follow-ups were excluded, which may lead to certain selection bias and limit the extrapolation of conclusions. Last, although this analysis covered an average of 5.3 years (range 2–7), it may not be long enough to evaluate the measurable change in cognitive function and the long-term influence of living arrangements as cognitive decline is a chronic process. Thus, we will continue to follow up on the latest data from CHARLS and explore these associations over a longer time.

In conclusion, this study found that older people living alone, living with adult children, living with both spouse and adult children and living with others all had a faster rate of cognitive decline than those living with spouse. Also, the relationship between living arrangements and cognitive decline varies by gender. The stereotype that living with children is beneficial to the health of older people deserves and needs to be challenged by more research. Although filial piety was valued in traditional China, modern Chinese society has become more individualized and less family-oriented, with spouses replacing children as the most significant family ties. Nevertheless, we still propose that adult children should provide their older parents with more emotional, caring and appropriate financial support. To slow cognitive decline with age, improve quality of life and promote successful ageing, the government should improve social security and community services and establish a variety of support systems for older people. Also, consideration needs to be given to how non-family care policies and programmes might be established to support older individuals who live alone. In addition, more strategies to prevent cognitive decline should be proposed that take into account gender differences. More in-depth research is needed to better understand the mechanisms underlying the role of living arrangements in cognitive decline with age in the future.

## Electronic supplementary material

Below is the link to the electronic supplementary material.

Supplementary Material 1.
Supplemental Methods.
1) Demographic characteristics.
2) Health status.
3) Child characteristics.
4) Socioeconomic level.
Supplementary tables.
Table S1. The questionnaire items of CESD-10 and its answer options and marks assigned.
Table S2. Baseline characteristics between participants included and not included.
Table S3 Sensitivity analysis of the association between living arrangements and cognitive decline.
Table S4 Sensitivity analysis of gender differences in the association between living arrangements and cognitive decline.

## Data Availability

The datasets used in this study are available on the CHARLS website at http://charls.pku.edu.cn/en.

## Abbreviations

CHARLS: China Health and Retirement Longitudinal Study.
IADLs: Instrumental activity of daily living.
SD: Standard deviation.
ref: The reference category.
