# Supplementary material for: Association between living arrangements and cognitive decline in older adults: A nationally representative longitudinal study in China
Source: BMC Geriatr. 2022 Nov 8;22:843. doi: 10.1186/s12877-022-03473-x (PMC9644618; doi:10.1186/s12877-022-03473-x)
Supplement: Supplementary file 1 — Supplementary Material 1. Supplemental Methods. 1) Demographic characteristics. 2) Health status. 3) Child characteristics. 4) Socioeconomic level. Supplementary tables. Table S1. The questionnaire items of CESD-10 and its answer options and marks assigned. Table S2. Baseline characteristics between participants included and not included. Table S3 Sensitivity analysis of the association between living arrangements and cognitive decline. Table S4 Sensitivity analysis of gender differences in the association between living arrangements and cognitive decline. [file 12877_2022_3473_MOESM1_ESM.docx]

**Association between living arrangements and cognitive decline in older adults: a nationally representative longitudinal study in China**

**Additional File**

Supplemental Methods

1) Demographic characteristics

2) Health status

3) Child characteristics

4) Socioeconomic level

Supplementary tables

Table S1. The questionnaire items of CESD-10 and its answer options and marks assigned

Table S2. Baseline characteristics between participants included and not included

Table S3 Sensitivity analysis of the association between living arrangements and cognitive decline

Table S4 Sensitivity analysis of gender differences in the association between living arrangements and cognitive decline

**Supplemental Methods**

**1.** **Demographic characteristics**

Age was classified into the following four groups: 60–64, 65–69, 70–74, and older than 75 years old.

Geographic residence indicates respondents’ household living region, which was divided into rural or urban based on the National Bureau of Statistics of the People's Republic of China.

In CHARLS, education levels were divided into eleven categories, including (1) no formal education (illiterate); (2) did not finish primary school but can read; (3) Sishu (private tutoring); (4) elementary/primary school; (5) middle school; (6) high school; (7) vocational school; (8) two-/three-year college/associate degree; (9) four-year college/Bachelor’s degree; (10) Master’s degree; (11) Doctoral degree/Ph.D. Due to the relatively low education level of older people in China (thirty-three per cent of participants had an educational level higher than primary school), we divided educational levels into four levels: no formal education, capable of reading and/or writing, primary school and middle school and above.

**2. Health status**

Physical comorbidity data included conditions for which respondents self-reported receiving a diagnosis from a physician, such as dyslipidemia, diabetes or high blood sugar, cancer or malignant tumor, chronic lung disease, liver disease, heart disease, stroke, kidney disease, stomach or digestive disease, arthritis or rheumatism, and asthma. The number of physical comorbidities was calculated and categorized as 0,1-2 and ≥ 3.

Feeling pain was self-reported via a question: “Are you often troubled with any body pains?”.

Instrumental activities of daily living (IADLs) was evaluated by the Lawton and Brody’s scale referring to doing housework, cooking, taking medicine, shopping, and taking care of finances. Respondents were asked to choose from the four corresponding answers for each item: (1) No, I do not have any difficulty, (2) I have difficulty but still can do it, (3) Yes, I have difficulty and need help, and (4) I cannot do it. Participants who reported having any difficulty in any items were classified as with IADLs impaired.

The Chinese version of 10-item Center for Epidemiologic Studies Depression (CESD-10) Scale was used to measure depressive symptoms, which reflected the respondents’ depressive symptoms experienced over the previous week. The ten items included three items on depressed mood, five items on somatic symptoms and two items on positive mood. Except for two items on positive emotions which were reverse scored, the other eight items were scored 0, 1, 2 and 3 according to their frequency of symptoms. The total CESD-10 score for the 10 items ranges from 0 to 30, with higher scores indicating more severe depressive symptoms. Participants with a CESD-10 score above 10 points were sorted as depressed. The questionnaire items for the CESD-10 and their answer options and score allocations are shown in **Table S1.**

**Table S1. The questionnaire items of CESD-10 and its answer options and marks assigned**

| Questionnaire items | Answer options and marks assigned | | | |
| --- | --- | --- | --- | --- |
| I was bothered by things that don’t usually bother me | 0=less 1 day | 1= 1-2 days | 2= 3-4 days | 3= 5-7 days |
| I had trouble keeping my mind on what I was doing | 0=less 1 day | 1= 1-2 days | 2= 3-4 days | 3= 5-7 days |
| I felt depressed | 0=less 1 day | 1= 1-2 days | 2= 3-4 days | 3= 5-7 days |
| I felt everything I did was an effort | 0=less 1 day | 1= 1-2 days | 2= 3-4 days | 3= 5-7 days |
| I felt hopeful about the future | 0=5-7 days | 1= 3-4 days | 2= 1-2 days | 3= less 1 day |
| I felt fearful | 0=less 1 day | 1= 1-2 days | 2= 3-4 days | 3= 5-7 days |
| My sleep was restless | 0=less 1 day | 1= 1-2 days | 2= 3-4 days | 3= 5-7 days |
| I was happy | 0=5-7 days | 1= 3-4 days | 2= 1-2 days | 3= less 1 day |
| I felt lonely | 0=less 1 day | 1= 1-2 days | 2= 3-4 days | 3= 5-7 days |
| I could not get “going” | 0=less 1 day | 1= 1-2 days | 2= 3-4 days | 3= 5-7 days |

The CHARLS questionnaire included eight categories of social activities, including: (1) Interacted with friends; (2) Played Ma-jong, played chess, played cards, or went to community club; (3) Provided help to family, friends, or neighbors who do not live with you; (4) Went to a sport, social, or other kind of club; (5) Took part in a community-related organization; (6) Done voluntary or charity work; (7) Cared for a sick or disabled adult who does not live with you; (8) Attended an educational or training course. Participation in social activities was defined as the respondent having participated in at least one of these social activities in the previous month.

**3. Child characteristics**

The number of adult children refers to the number of alive children of respondents aged ≥22 years who are not in school. The number was classified into three categories: 1, 2-3 and ≥4.

The average of years of schooling of adult children is the ratio of the years of schooling of all adult children to the number of adult children. The average years of schooling of adult children was centered using the average value.

**4** **Socioeconomic characteristics**

Average annual household expenditure per capita was calculated by taking total household consumption divided by the number of people in the household. Total household consumption comprised of a wide range of expenditures, which was the sum of food consumption and non-food consumption. To capture the non-linear relationship between income and outcome variables, the average annual household expenditure was log-transformed in the analysis.

**Table S2. Baseline characteristics between participants included and not included**

| Characteristics | Included (n=6074) | Excluded (n=4245) | *P* value |
| --- | --- | --- | --- |
| **Cognitive function, mean ± SD** | 9.93±4.26 | 9.65±4.61 | <0.001 |
| **The living arrangement, n (%)** |  |  | <0.001 |
| Living alone | 482 (7.94) | 441 (10.41) |  |
| Living with spouse | 2728 (44.91) | 1800 (42.49) |  |
| Living with adult children | 639 (10.52) | 483 (11.40) |  |
| Living with spouse and adult children | 2103 (34.62) | 1385 (32.70) |  |
| Living with others | 122 (2.01) | 127 (3.00) |  |
| **Age (years at baseline), mean ± SD** | 67.24±5.99 | 64.67±7.34 | <0.001 |
| **Gender, n (%)** |  |  | 0.213 |
| Male | 3016 (49.65) | 2054 (48.41) |  |
| Female | 3058 (50.35) | 2189 (51.59) |  |
| **Geographic residence, n (%)** |  |  | 0.600 |
| Rural | 3627 (59.71) | 2513 (59.20) |  |
| Urban | 2447 (40.29) | 1732 (40.80) |  |
| **Education, n (%)** |  |  | <0.001 |
| No formal education | 1729 (28.47) | 1476 (36.71) |  |
| Capable of reading and/or writing | 1300 (21.40) | 836 (20.79) |  |
| Primary school | 1600 (26.34) | 752 (18.71) |  |
| Middle school and above | 1445 (23.79) | 957 (23.79) |  |
| **Working status, n (%)** |  |  | <0.001 |
| No | 2710 (44.62) | 1672 (40.40) |  |
| Yes | 3364 (55.38) | 2467 (59.60) |  |
| **The number of physical comorbidities, n (%)** |  |  | <0.001 |
| 0 | 1858 (30.59) | 2243 (53.61) |  |
| 1-2 | 2972 (48.93) | 1410 (33.70) |  |
| ≥3 | 1244 (20.48) | 531 (12.69) |  |
| **Feeling pain, n (%)** |  |  | 0.090 |
| No | 4050 (66.68) | 2683 (68.30) |  |
| Yes | 2024 (33.32) | 1245 (31.70) |  |
| **IADLs, n (%)** |  |  | <0.001 |
| Unimpaired | 4630 (76.23) | 2885 (71.69) |  |
| Impaired | 1444 (23.77) | 1139 (28.31) |  |
| **Depressive symptoms, n (%)** |  |  | 0.301 |
| No | 3854 (63.45) | 2163 (62.39) |  |
| Yes | 2220 (36.55) | 1304 (37.61) |  |
| **Social activity participation, n (%)** |  |  | 0.008 |
| No | 3198 (52.65) | 2033 (55.40) |  |
| Yes | 2876 (47.35) | 1637 (44.60) |  |
| **Number of adult children, n (%)** |  |  | <0.001 |
| 1 | 582 (9.58) | 524 (16.90) |  |
| 2-3 | 3041 (50.07) | 2400 (77.39) |  |
| ≥4 | 2451 (40.35) | 177 (5.71) |  |
| **Average schooling year of children (Centered), mean ± SD** | 8.41±3.60 | 9.06±3.65 | <0.001 |
| **Household expenditure per capita(log), mean ± SD** | 8.54±0.94 | 8.39±0.93 | <0.001 |

Note: (1) Except for the variable cognitive function, all other variables were measured at baseline interview. (2) Sex differences in continuous variables were tested using t-tests and in categorical variables using chi-square tests. (3) The results were unweighted. (4) SD = standard deviation, IADLs = instrumental activity of daily living*.*

**Table S3 Sensitivity analysis of the association between living arrangements and cognitive decline**

|  | **Model 1** | **Model 2** | **Model 3** |
| --- | --- | --- | --- |
| **Fixed Effects** |  |  |  |
| Intercept | 11.190^***^ | 3.592^***^ | 3.514^***^ |
| Living arrangement  (ref=living with spouse) |  |  |  |
| Living alone | -0.665^***^ | -0.298^**^ | -0.077 |
| Living with adult children | -1.188^***^ | -0.269^*^ | -0.016 |
| Living with spouse and adult children | -0.596^***^ | -0.055 | 0.021 |
| Living with others | -1.253^***^ | -0.159 | 0.166 |
| Time (years since baseline) | -0.229^***^ | -0.224^***^ | -0.192^***^ |
| Age (ref=60-64, at baseline) |  |  |  |
| 65-69 | -0.599^***^ | -0.386^***^ | -0.389^***^ |
| 70-74 | -2.048^***^ | -1.178^***^ | -1.179^***^ |
| ≥75 | -4.196^***^ | -2.460^***^ | -2.464^***^ |
| Gender (ref= Male) |  |  |  |
| Female | -2.328^***^ | -0.633^***^ | -0.633^***^ |
| Geographic residence (ref= Rural) |  |  |  |
| Urban | 2.422*** | 0.156^*^ | 0.156^*^ |
| Education  (ref= No formal education) |  |  |  |
| Capable of reading and/or writing |  | 2.645^***^ | 2.643^***^ |
| Primary school |  | 3.978^***^ | 3.977^***^ |
| Middle school and above |  | 4.957^***^ | 4.957^***^ |
| Working status (ref= No) |  |  |  |
| Yes |  | 0.066 | 0.066 |
| The number of physical comorbidities (ref=0) |  |  |  |
| 1-2 |  | 0.193^**^ | 0.192^**^ |
| ≥3 |  | 0.409^***^ | 0.409^***^ |
| Feeling pain (ref=No) |  |  |  |
| Yes |  | -0.291^***^ | -0.292^***^ |
| IADLs (ref=Unimpaired) |  |  |  |
| Impaired |  | -0.928^***^ | -0.929^***^ |
| Depressive symptoms (ref=No) |  |  |  |
| Yes |  | -0.411^***^ | -0.411^***^ |
| Social activity participation (ref=No) |  |  |  |
| Yes |  | 0.642 ^***^ | 0.641 ^***^ |
| Number of adult children (ref=1) |  |  |  |
| 2-3 |  | 0.135 | 0.134 |
| ≥4 |  | 0.098 | 0.097 |
| Average years of schooling of adult children (centered) |  | 0.191^***^ | 0.191^***^ |
| Household expenditure per capita (log) |  | 0.503^***^ | 0.504^***^ |
| Time * Living alone |  |  | -0.086^**^ |
| Time * Living with adult children |  |  | -0.099^***^ |
| Time * Living with spouse and adult children |  |  | -0.030 |
| Time * Living with others |  |  | -0.126^**^ |
| **Random Effects** |  |  |  |
| Level 2: Individual |  |  |  |
| Individual -variance | 11.434^***^ | 5.403^***^ | 5.404^***^ |
| Level 1: Point in time |  |  |  |
| Point in time-variance | 7.958^***^ | 8.028^***^ | 8.019^***^ |

Note: (1) Sensitivity analysis using a full sample after multiple imputation with 10176 individuals consisting of 32171 observations. (2) ref = the reference category, IADLs = instrumental activity of daily living. (3) ^*^P < 0.05, ^**^P < 0.01, ^***^P < 0.001*.*

**Table S4 Sensitivity analysis of gender differences in the association between living arrangements and cognitive decline**

|  | **Males**  **(n=4996)** | **Females**  **(n=5180)** |
| --- | --- | --- |
| **Fixed Effects** |  |  |
| Intercept | 3.202^***^ | 3.419^***^ |
| Living arrangement  (ref=Living with spouse) |  |  |
| Living alone | -0.190 | -0.039 |
| Living with adult children | -0.028 | -0.054 |
| Living with spouse and adult children | 0.020 | 0.003 |
| Living with others | 0.309 | 0.043 |
| Time (years since baseline) | -0.172^***^ | -0.214^***^ |
| Age (ref=60-64, at baseline) |  |  |
| 65-69 | -0.255^***^ | -0.543^***^ |
| 70-74 | -1.122^***^ | -1.302^***^ |
| ≥75 | -2.474^***^ | -2.509^***^ |
| Geographic residence (ref= Rural) |  |  |
| Urban | 0.027 | 0.317^**^ |
| Education  (ref= No formal education) |  |  |
| Capable of reading and/or writing | 2.356^***^ | 2.698^***^ |
| Primary school | 3.653^***^ | 4.078^***^ |
| Middle school and above | 4.583^***^ | 5.271^***^ |
| Working status (ref= No) |  |  |
| Yes | 0.223^*^ | -0.080 |
| The number of physical comorbidities (ref=0) |  |  |
| 1-2 | 0.270^**^ | 0.130 |
| ≥3 | 0.373^**^ | 0.439^***^ |
| Feeling pain (ref=No) |  |  |
| Yes | -0.415^***^ | -0.196^*^ |
| IADLs (ref=Unimpaired) |  |  |
| Impaired | -1.035^***^ | -0.841^***^ |
| Depressive symptoms (ref=No) |  |  |
| Yes | -0.410^***^ | -0.388^***^ |
| Social activity participation (ref=No) |  |  |
| Yes | 0.700^***^ | 0.594^***^ |
| Number of adult children (ref=1) |  |  |
| 2-3 | 0.204 | 0.022 |
| ≥4 | 0.223 | -0.027 |
| Average schooling year of children (centered) | 0.167^***^ | 0.213^***^ |
| Household expenditure per capita (log) | 0.551^***^ | 0.454^**^ |
| Time * Living alone | -0.147^**^ | -0.041 |
| Time * Living with adult children | -0.112^*^ | -0.080^**^ |
| Time * Living with spouse and adult children | -0.028 | -0.036^*^ |
| Time * Living with others | -0.084 | -0.142^**^ |
| **Random Effects** |  |  |
| Level 2: Individual |  |  |
| Individual -variance | 4.957^***^ | 5.730^***^ |
| Level 1: Point in time |  |  |
| Point in time-variance | 8.523^***^ | 7.530^***^ |

Note: (1) Sensitivity analysis using a full sample after multiple imputation with 4996 males consisting of 15767 observations and 5180 females consisting of 16404 observations. (2) ref = the reference category, IADLs = instrumental activity of daily living. (3) ^*^P < 0.05, ^**^P < 0.01, ^***^P < 0.001.
